# Hospital change to mixed lipid emulsion from soybean oil-based lipid emulsion for parenteral nutrition in hospitalized and critically ill adults improves outcomes: a pre–post-comparative study

**DOI:** 10.1186/s13054-022-04194-8

**Published:** 2022-10-18

**Authors:** Krista L. Haines, Tetsu Ohnuma, Charles Trujillo, Obanor Osamudiamen, Vijay Krishnamoorthy, Karthik Raghunathan, Paul E. Wischmeyer

**Affiliations:** 1 Division of Trauma and Critical Care and Acute Care Surgery, Department of Surgery, Duke University Medical Center, Durham, NC USA; 2 The Critical Care and Perioperative Epidemiologic Research (CAPER) Unit, Duke University Medical Center, Durham, NC USA; 3 Department of Anesthesiology, Duke University School of Medicine, Duke University Medical Center (DUMC), Mail # 41, 2301 Erwin Road, 5692 HAFS, Box 3094, Durham, NC 27710 USA

**Keywords:** SMOFLipid, TPN, PN, Critical illness, Nutrition, Patient outcomes, ICU, Lipids

## Abstract

**Introduction:**

Early data suggest use of a mixed lipid emulsion (LE) with a soybean oil reduction strategy in parenteral nutrition (PN) may improve clinical outcomes. Duke University Hospital made a full switch to a Soybean oil/MCT/Olive/Fish Oil lipid (4-OLE) from pure soybean oil-based LE (*Intralipid*, Baxter Inc) in May 2017. Since 4-OLE has limited evidence related to its effects on clinical outcome parameters in US hospitals, evidence for clinical benefits of switching to 4-OLE is needed. Therefore, we examined the clinical utility of a hospital-wide switch to 4-OLE and its effect on patient outcomes.

**Methods:**

We conducted a single-center retrospective cohort study among adult patients (> 18 years) requiring PN from 2016 to 2019. Our primary exposure was treatment period (1-year pre-4-OLE switch versus 2-year post). We used multivariable regression models to examine our primary outcomes, the association of treatment period with hospital length of stay (LOS), and secondary outcomes liver function, infections, and ICU LOS. Analyses were stratified into critically ill and entire adult cohort.

**Results:**

We identified 1200 adults hospitalized patients. 28% of PN patients (*n* = 341) were treated pre-4-OLE switch and 72% post-4-OLE (*n* = 859). In the adult cohort, 4-OLE was associated with shorter hospital LOS (IRR 0.97, 95% CI 0.95–0.99, *p* = 0.039). The ICU cohort included 447 subjects, of which 25% (*n* = 110) were treated pre-4-OLE switch and 75% (*n* = 337) were post-switch. ICU patients receiving 4-OLE were associated with shorter hospital LOS (IRR 0.91, 95% CI 0.87–0.93, *p* < 0.0001), as well as a shorter ICU LOS (IRR 0.90, 95% CI 0.82–0.99, *p* = 0.036). 4-OLE ICU patients also had a significantly lower delta total bilirubin (− 1.6, 95% CI − 2.8 to − 0.2, *p* = 0.021) and reduced urinary tract infection (UTI) rates (OR 0.50, 95% CI 0.26–0.96, *p* = 0.038). There were no associations in AST, ALT, or total bilirubin in ICU and all adult patients.

**Conclusion:**

4-OLE was successfully implemented and reduced soybean oil LE exposure in a large academic hospital setting. The introduction of 4-OLE was associated with reduced LOS, UTI rates, and mitigated hepatic dysfunction in critically ill patients. Overall, these findings prove a switch to a soybean oil-LE sparing strategy using 4-OLE is feasible and safe and is associated with improved clinical outcomes in adult PN patients.

## Introduction

Moderate-to-severe malnutrition is associated with an increased risk of mortality, infections, hospital length of stay, and higher healthcare-associated costs, especially in critically ill patients [1–5]. Historically, the use of various modalities to combat malnutrition in critical illness has primarily consisted of enteral and parenteral based nutritional support regimens. Parenteral nutrition (PN) is advocated for when enteral nutrition (EN) cannot be administered, such as those with recent abdominal surgery and/or GI intolerance, and is therefore recommended by current American Society for Parenteral and Enteral Nutrition (ASPEN) guidelines [6]. However, the use of PN, specifically the composition of traditional lipid emulsions (LE), is suggested to have detrimental effects on critically ill patients.

Pure soybean oil-based LEs have been predominantly used in the USA and have been implicated in the adverse outcomes related to PN. The use of soybean oil-based LE has been shown to propagate a pro-inflammatory cascade and increase reactive oxidative stress [7–9]. In severe critical illness, soybean oil-based LE has been shown to impair cellular-mediated immunity with amplification of IL-6 levels [9, 10]. Additionally, soybean oil-based emulsions are known to impair chemotaxis and phagocytotic ability, increasing bacteremia and infection risks [9, 11–14]. Traditionally, soybean oil-based LE also worsens hepatic dysfunction and cholestasis risks [15].

The negative effects seen with soybean oil-based emulsions are thought to be largely due to the high content of long-chain triglycerides and omega-6 fatty acids, inherent with pro-inflammatory properties. Given the issues surrounding traditional soybean oil-based LE, the use of “soybean oil-sparing” or “omega-6 sparing” strategies has come to the forefront of nutritional support in critical care [16, 17]. These strategies include mixed formulations with various compositions of medium-chain triglycerides, fish oil, and/or olive oil-based emulsions [16, 17]. The rationale behind this strategy is to increase the percentage of the key beneficial omega-3 fatty acids of EPA (eicosapentaenoic acid) and DHA (docosahexaenoic acid) that have beneficial immunomodulating effects, including reduced inflammatory cytokine production [7, 18]. The benefit of EPA and DHA supplementation is due to active metabolites called resolvins, limiting inflammation and additional end-organ injury [19]. A systematic review found that soybean oil-reducing strategies, including use of lipid preparations with increased amounts of EPA and DHA containing lipids, may decrease intensive care unit (ICU) length of stay (LOS), hospital LOS, and time on the ventilator [20]. This same analysis failed to demonstrate any benefit in mortality with this strategy [20]. Related literature coincides with a lack of conclusive evidence to support soybean oil reduction strategies or their benefit on mortality [21–23].

Despite the controversy and paucity of the literature to support its use, “soybean oil-sparing” strategies are recommended for patients requiring PN [24, 25]. Of these varieties, one specific mixed-oil-based LE that has demonstrated increased interest and use outside of the USA is SMOFLipid (4-OLE), which is a mixture of 3 g soybean oil, 3 g medium-chain triglycerides, 2.5 g olive oil, 1.5 g fish oil, 200 mg alpha-tocopherol and 47.6 mg phytosterols per 100 ml of emulsion [26]. This type of LE contains 15% of the lipid as fish oil which contains 30% of the fish oil as EPA and DHA. Thus, this 4-OLE lipid has a 5% EPA + DHA acid composition which may convey potential anti-inflammatory properties discussed previously [19, 26]. A recent meta-analysis found significant benefits to LEs which reduce soybean oil content (and contain added EPA + DHA) showing reduced rates of sepsis, infection, and length of stay [27]. However, this study failed to show mortality benefits using soybean oil-reducing with added EPA/DHA-based LE supplementation [27]. Duke University Health System recently implemented 4-OLE as part of its PN regimen. Given the lack of evidence related to 4-OLE use and its relative infancy as an option for LE supplementation, the objective of this study was to investigate the clinical effect of 4-OLE supplementation after widespread implementation in an adult hospital population.

## Methods

### Study design and population

We conducted a retrospective cohort study using electronic health record data from 2016 to 2018. This study was reviewed by the Duke Institutional and Ethics Review Board and was granted approval (Approval Number: Pro00102602). In the pure soybean oil-based group (IL), all adult patients receiving PN with any lipid delivery from May 16, 2016–May 16, 2017, were included in analysis. This constituted one year prior to the full switch over from IL to 4-OLE at Duke University Hospital in all PN patients without contraindications (i.e., allergy) on May 16, 2017. The 4-OLE group consisted of all adult patients receiving PN with SMOFLipid from May 17, 2017–September, 1, 2019. Patients after May 17, 2016, who received IL due to an allergy to a 4-OLE component were excluded from analysis. Only 1 year prior to switch to 4-OLE was chosen to minimize effects of any potential changes in clinical practice on outcomes. Inclusion criteria were adults, defined as age ≥ 18 years of age, who required PN initiation for adequate goal nutrition. A subgroup analysis was performed those who required an ICU level of care.

The following data were collected for each patient: age, sex, race, ethnicity, body mass index (BMI), insurance status, ICU location, emergency department (ED) admission, ICU admission, malnutrition at admission, comorbidities using the binary Elixhauser’s comorbidity indicators [28], PN utilization profile (type of lipid emulsion and dose of lipids, protein, dextrose in g/kg/d, duration), hospital outcomes (ICU length of stay, hospital length of stay, in-hospital mortality, 30-day readmission, and 90-day readmission), infections (pneumonia, urinary tract infection, and all infections), mechanical ventilation, statin use, insulin use, antibiotic use, laboratory values (aspartate transaminase (AST), alanine transaminase (ALT), alkaline phosphatase (ALP), total bilirubin, blood glucose, C-reactive protein, and triglycerides). Comorbidities and infections were identified using the International Classification of Diseases, Tenth Revision (ICD-10) diagnosis codes.

### Exposure

Patients from 2016 to May 2017 prior to the change (*Intralipid* (IL) (Baxter Inc, Deerfield, IL, USA) group) were compared with patients from May 2017 to 2019 after the clinical change (4-OLE group).

### Outcomes

The primary outcome was hospital LOS. Secondary outcomes included hospital mortality, 30-day readmission, ICU LOS, all-cause infections, calorie delivery, liver function tests (AST, ALT, ALP, and total bilirubin). We calculated delta values by subtracting initial values from max values.

### Covariates

The following covariates were used in the analysis: age, sex, race, ethnicity, insurance status, cancer, malnutrition, ED admission, ICU admission, and BMI.

### Statistical analysis

Descriptive statistics were calculated and presented as number (percentage) for categorical variables and median (interquartile range) or mean ± standard deviation (SD) for continuous variables. Continuous variables were compared using an unpaired *t* test or a Wilcoxon test. Pearson’s chi-squared test or Fisher’s exact test was used for categorical data.

We used multivariable Poisson regression models to analyze the association between 4-OLE and LOS. Coefficients in the Poisson model were exponentiated to obtain incidence rate ratios (IRRs). We used multivariable linear regression models for nutrition delivery variables and liver functions tests. We used multivariable logistic regression models for hospital mortality and 30-day readmission. All covariates above were included in the models. We stratified the cohort to patients admitted to ICU in the sensitivity analysis and performed the same analysis mentioned above except for covariates. (ICU admission was not included in the covariates.) We performed the complete case analysis—the type I error rate was set at 0.05 as the threshold for statistical significance. Statistical analyses were performed using SAS version 9.4 (SAS Institute; Cary, NC).

## Results

### Demographic and clinical characteristics in adults

We identified 1200 adults. 28% of PN patients (*n* = 341) received *Intralipid* (Baxter Inc, Deerfield, IL, USA (IL)) pre-switch and 72% received 4-OLE (*n* = 859). Median age of the overall cohort (58.5 years old, range 44–68 years, *p* = 0.049) was statistically different with the IL group having a slightly younger population (56 years old, range 42–67, *p* = 0.049) in Table 1. There was no significant difference in BMI (25.5 kg/m^2^, 21.6–29.6, *p* = 0.81) or in sex distribution between both cohorts. Baseline liver function tests were similar between the two groups. However, the 4-OLE group had significantly smaller number of patients with renal failure (10.4% vs 16.7%, *p* = 0.002), congestive heart failure (CHF) (8.7%vs 13.5%, *p* = 0.014), and underlying malignancy (18.4% vs 26.1%, *p* = 0.003).

**Table 1:** Baseline characteristics among all adults

	Total	Intralipid*	4-OLE	*p* value
	(*N* = 1200)	(*N* = 341)	(*N* = 859)	
Age (in years), median (IQR)	58.5 (44.0–68.0)	56.0 (42.0–67.0)	59.0 (46.0–68.0)	0.049
Male	635 (52.92%)	188 (55.13%)	447 (52.04%)	0.33
Race				
Caucasian/white	764 (63.67%)	225 (65.98%)	539 (62.75%)	0.31
Black or African-American	333 (27.75%)	93 (27.27%)	240 (27.94%)	
Other	103 (8.58%)	23 (6.74%)	80 (9.31%)	
Hispanic	44 (3.67%)	10 (2.93%)	34 (3.96%)	0.39
Payor				
MCO	373 (31.08%)	101 (29.62%)	272 (31.66%)	0.87
Medicaid	99 (8.25%)	30 (8.80%)	69 (8.03%)	
Medicare	568 (47.33%)	162 (47.51%)	406 (47.26%)	
Other	160 (13.33%)	48 (14.08%)	112 (13.04%)	
Comorbidity at admission				
Malnutrition	788 (65.67%)	226 (66.28%)	562 (65.42%)	0.78
Congestive heart failure	121 (10.08%)	46 (13.49%)	75 (8.73%)	0.014
Cancer	247 (20.58%)	89 (26.10%)	158 (18.39%)	0.003
Chronic pulmonary disease	140 (11.67%)	49 (14.37%)	91 (10.59%)	0.07
Liver disease	108 (9.00%)	37 (10.85%)	71 (8.27%)	0.16
Renal failure	146 (12.17%)	57 (16.72%)	89 (10.36%)	0.002
Diabetes mellitus	189 (15.75%)	59 (17.30%)	130 (15.13%)	0.35
Body weight, kg, median (IQR)	74.7 (61.0–88.2)	71.8 (60.5–87.1)	72.4 (60.6–87.4)	0.37
BMI (kg/m^2^), median (IQR)	25.5 (21.6–29.6)	25.4 (21.4–29.2)	25.5 (21.7–29.6)	0.81
ED admission	452 (37.67%)	116 (34.02%)	336 (39.12%)	0.1
ICU admission	447 (37.25%)	110 (32.26%)	337 (39.23%)	0.024
Mechanical ventilation	223 (18.58%)	59 (17.30%)	164 (19.09%)	0.47
Antibiotic use	1088 (90.67%)	312 (91.50%)	776 (90.34%)	0.53
Use of insulin	582 (48.50%)	173 (50.73%)	409 (47.61%)	0.33
Use of statin	220 (18.33%)	56 (16.42%)	164 (19.09%)	0.28

*IQR* Interquartile range; *MCO* Managed care organization; *BMI* body mass index in kg/m^2^; *ED* emergency department; *ICU* intensive care unit; and *4-OLE* Soy/MCT/Olive/Fish Oil lipid

^*^Intralipid—(Baxter Inc, Deerfield, IL, USA)

Nutrition profiles and liver function tests are summarized in Table 2. 4-OLE was associated with higher ALP (29.0, 95% CI 6.2–51.8, *p* = 0.013). 4-OLE use was also associated with higher daily lipid dosage (0.06, 95% CI 0.01–0.11, *p* = 0.018).

**Table 2:** Unadjusted and adjusted analysis for nutrition profiles and liver function tests among all adults

	Intralipid*	4-OLE	Unadjusted analysis	Adjusted analysis	
	(*N* = 341)	(*N* = 859)	*p* value	Estimate (95% CI)	*p*-value
Total days receiving lipids, median (IQR)	8.0 (4.0–12.5)	7.0 (3.0–15.0)	0.62	1.49 (0.11, 2.87)	0.034
Daily lipid dosage (g/kg/day), median (IQR)	0.6 (0.4–0.7)	0.6 (0.4–0.8)	0.041	0.03 (− 0.01, 0.06)	0.11
Daily calories (calories/kg/day), median (IQR)	23.7 (18.8–28.9)	24.8 (19.3–30.6)	0.1	0.18 (− 0.02, 0.37)	0.076
Daily protein (g/kg/day), median (IQR)	0.8 (0.7–1.0)	0.9 (0.7–1.0)	0.06	0.02 (− 0.00, 0.05)	0.0763
Daily dextrose (g/kg/day), median (IQR)	1.5 (1.1–1.9)	1.5 (1.1–2.0)	0.45	0.04 (− 0.04, 0.11)	0.3104
*Liver function tests, median (IQR)*					
Delta AST (units/L)	15.0 (2.0–42.0)	16.0 (2.0–52.5)	0.4	61.9 (− 62.1, 185.8)	0.33
Delta ALT (units/L)	13.0 (0.5–44.5)	16.0 (1.0–54.5)	0.23	44.9 (− 8.6, 98.4)	0.1
Delta ALP (international units/L)	27.0 (3.0–63.0)	26.5 (0.0–104.0)	0.25	29.0 (6.2, 51.8)	0.013
Delta total bilirubin (mg/dL)	0.5 (0.2–1.3)	0.6 (0.1–1.4)	0.85	− 0.4 (− 1.0, 0.2)	0.21

We adjusted for age, sex, race, ethnicity, insurance status, cancer, malnutrition, ED admission, ICU admission, and BMI in kg/m^2^. The reference was the 4-OLE group

*IQR* Interquartile range; *4-OLE* SMOFLipid

^*^Intralipid—(Baxter Inc, Deerfield, IL, USA)

### Association of switch to 4-OLE with clinical outcomes

In adult patients, crude outcomes are summarized in Table 3. In multivariable regression models, 4-OLE was associated with a reduced LOS [incident rate ratio (IRR) 0.97, 95% CI 0.95–0.99, *p* = 0.039] (Table 3). However, overall infectious complications of any kind nor hospital mortality were different between groups. There was also no significant difference in 30 and 90 day readmission rates.

**Table 3:** Associations of 4-OLE with outcomes among all adults

	Intralipid*	4-OLE	Unadjusted analysis	Adjusted analysis	
	(*N* = 341)	(*N* = 859)	*p* value	IRR (95% CI)	*p*-value
LOS, median (IQR)	22.2 (13.1–35.2)	20.3 (11.7–34.1)	0.28	0.97 (0.95, 0.99)	0.039

	Intralipid*	4-OLE	Unadjusted analysis	Adjusted analysis	
	(*N* = 341) (%)	(*N* = 859) (%)	*p* value	OR (95% CI)	*p*-value
Hospital mortality	32 (9.38)	102 (11.87)	0.22	1.13 (0.72, 1.78)	0.6
30 day readmission	101 (29.62)	254 (29.57)	0.99	1.12 (0.83, 1.49)	0.46
90 day readmission	237 (69.50)	569 (66.24)	0.28	0.97 (0.73, 1.30)	0.85
Any infection	153 (44.87)	364 (42.37)	0.43	0.82 (0.63, 1.08)	0.16
Pneumonia	39 (11.44)	103 (11.99)	0.79	0.93 (0.61, 1.41)	0.73
UTI	33 (9.68)	62 (7.22)	0.15	0.69 (0.44, 1.10)	0.12

We adjusted for age, sex, race, ethnicity, insurance status, cancer, malnutrition, ED admission, ICU admission, and BMI in kg/m^2^

*LOS* Length of stay, *IRR* Incident rate ratio, *OR* Odds ratio, *UTI* Urinary tract infection, *CI* Confidence interval; *4-OLE* SMOFLipid

^*^Intralipid—(Baxter Inc, Deerfield, IL, USA)

### Demographic and clinical characteristics in critically ill patients

The ICU cohort included 447 total subjects, of which 25% (*n* = 110) received *Intralipid* (Baxter Inc, Deerfield, IL, USA) (IL) and 75% (*n* = 337) received 4-OLE (Table 4). The median age (61 years old, 47–70) and BMI (26.8 kg/m^2^, 22.1–30.9) for the total cohort were similar between both groups. 4-OLE patients were less likely to have liver disease (9.2% vs 16.4%, *p* = 0.03) and renal failure (13.1% vs 29.1%, *p* < 0.001). Statin use was significantly greater in the 4-OLE cohort, and insulin use was higher in the IL cohort. Baseline liver function tests were found to be similar in both groups.

**Table 4:** Baseline characteristics among adults in intensive care unit

	Total	Intralipid*	4-OLE	*p* value
	(*N* = 447)	(*N* = 110)	(*N* = 337)	
Age (in years), median (IQR)	61.0 (47.0–70.0)	60.0 (48.0–72.0)	61.0 (46.0–69.0)	0.59
Male	255 (57.0%)	61 (55.5%)	194 (57.6%)	0.7
Race				
Caucasian/white	275 (61.5%)	72 (65.5%)	203 (60.2%)	0.09
Black or African-American	127 (28.4%)	33 (30.0%)	94 (27.9%)	
Other	45 (10.1%)	5 (4.5%)	40 (11.9%)	
Hispanic	13 (2.9%)	0 (0.0%)	13 (3.9%)	0.045
Payor				
MCO	126 (28.2%)	29 (26.4%)	97 (28.8%)	0.77
Medicaid	37 (8.3%)	7 (6.4%)	30 (8.9%)	
Medicare	227 (50.8%)	59 (53.6%)	168 (49.9%)	
Other	57 (12.8%)	15 (13.6%)	42 (12.5%)	
Comorbidity at admission				
Malnutrition	327 (73.2%)	88 (80.0%)	239 (70.9%)	0.06
Congestive heart failure	56 (12.5%)	18 (16.4%)	38 (11.3%)	0.16
Cancer	86 (19.2%)	28 (25.5%)	58 (17.2%)	0.06
Chronic pulmonary disease	72 (16.1%)	23 (20.9%)	49 (14.5%)	0.11
Liver disease	49 (11.0%)	18 (16.4%)	31 (9.2%)	0.03
Renal failure	76 (17.0%)	32 (29.1%)	44 (13.1%)	< 0.001
Diabetes mellitus	88 (19.7%)	27 (24.5%)	61 (18.1%)	0.14
Body weight, kg, median (IQR)	77.8 (61.4–98.6)	75.8 (63.0–91.4)	76.2 (62.5–91.9)	0.74
BMI (kg/m^2^), median (IQR)	26.8 (22.1–30.9)	26.9 (21.8–31.3)	26.6 (22.2–30.9)	0.83
ED admission	181 (40.5%)	36 (32.7%)	145 (43.0%)	0.06
Mechanical ventilation	223 (49.9%)	59 (53.6%)	164 (48.7%)	0.37
Antibiotic use	444 (99.3%)	110 (100.0%)	334 (99.1%)	1
Use of insulin	294 (65.8%)	81 (73.6%)	213 (63.2%)	0.045
Use of statin	106 (23.7%)	18 (16.4%)	88 (26.1%)	0.037

*IQR* Interquartile range; *MCO* managed care organization; *BMI* body mass index in kg/m^2^; *ED* emergency department; and *4-OLE* Soy/MCT/Olive/Fish Oil lipid

^*^Intralipid—(Baxter Inc, Deerfield, IL, USA)

Table 5 provides details regarding nutrition profiles and liver function tests in the ICU cohort. 4-OLE was not associated with total bilirubin, AST, ALT, ALP, or TG. 4-OLE use was also associated with higher daily lipid dosage (0.06, 95% CI 0.01 to 0.11, *p* = 0.018) (Table 5).

**Table 5:** Unadjusted and adjusted analysis for nutrition profiles and liver function tests among adults in intensive care unit

	Intralipid*	4-OLE	Unadjusted analysis	Adjusted analysis	*p*-value
	(*N* = 110)	(*N* = 337)	*p* value	Estimate (95% CI)	
Total days receiving lipids, median (IQR)	10.0 (6.0–15.0)	9.0 (5.0–19.0)	0.91	2.05 (− 0.64, 4.73)	0.13
Daily lipid dosage (g/kg/day), median (IQR)	0.5 (0.4–0.7)	0.6 (0.4–0.7)	0.08	0.06 (0.01, 0.11)	0.018
Daily calories (calories/kg/day), median (IQR)	21.5 (17.2–27.7)	23.1 (18.7–29.6)	0.08	0.26 (− 0.06, 0.59)	0.11
Daily protein (g/kg/day), median (IQR)	0.8 (0.7–0.9)	0.8 (0.7–1.0)	0.041	0.03 (− 0.01, 0.08)	0.11
Daily dextrose (g/kg/day), median (IQR)	1.4 (1.0–1.8)	1.4 (1.0–1.9)	0.44	0.05 (− 0.07, 0.16)	0.46
*Liver function tests, median (IQR)*					
Delta AST (units/L)	27.0 (8.0–99.0)	27.5 (8.0–99.0)	0.85	45.3 (− 201.1, 291.8)	0.72
Delta ALT (units/L)	26.0 (4.0–78.0)	24.0 (5.0–97.0)	0.79	41.7 (− 60.4, 143.7)	0.42
Delta ALP (international units/L)	56.0 (17.0–153.0)	47.0 (10.0–160.0)	0.78	11.6 (− 28.5, 51.7)	0.57
Delta total bilirubin (mg/dL)	1.1 (0.5–3.2)	1.0 (0.4–2.5)	0.28	− 1.6 (− 2.9, − 0.2)	0.021

We adjusted for age, sex, race, ethnicity, insurance status, cancer, malnutrition, ED admission, and BMI in kg/m^2^

*IQR* Interquartile range; *4-OLE* SMOFLipid

^*^Intralipid—(Baxter Inc, Deerfield, IL, USA)

### Association of switch to 4-OLE with clinical outcomes in critically ill patients

The crude outcomes are shown in Table 6. In multivariable regression models, we observed significant findings in comparing IL to 4-OLE patients in the ICU adult cohort with a significantly shorter hospital LOS (IRR 0.91, 95% CI 0.87–0.94, *p* < 0.0001) as well as a shorter ICU LOS (IRR 0.90, 95% CI 0.82–0.99, *p* = 0.036) (Table 6). 4-OLE was associated with reduced urinary tract infections (UTI) (odds ratio 0.50, 95% CI 0.26–0.96, *p* = 0.038). 4-OLE use was not associated with overall infections, hospital mortality, nor 30- or 90-day readmission rates.

**Table 6:** Associations of 4-OLE with outcomes among adults in intensive care unit

	Intralipid^*^	4-OLE	Unadjusted analysis	Adjusted analysis	*p*-value
	(*N* = 110)	(*N* = 337)	*p* value	IRR (95% CI)	
LOS, median (IQR)	32.7 (22.2–46.2)	27.8 (16.4–45.7)	0.013	0.91 (0.87, 0.94)	< 0.0001
ICU LOS, median (IQR)	3.5 (1.0–6.0)	2.0 (1.0–6.0)	0.08	0.90 (0.82, 0.99)	0.036

	Intralipid^*^	4-OLE	Unadjusted analysis	Adjusted analysis	*p*-value
	(*N* = 110) (%)	(*N* = 337) (%)	*p* value	OR (95% CI)	
Hospital mortality	22 (20.0)	75 (22.3)	0.62	1.17 (0.66, 2.06)	0.6
30 day readmission	30 (27.3)	65 (19.3)	0.08	0.61 (0.36, 1.04)	0.067
90 day readmission	63 (57.3)	185 (54.9)	0.66	0.88 (0.55, 1.39)	0.58
Any infection	66 (60.0)	197 (58.5)	0.78	0.90 (0.56, 1.43)	0.65
Pneumonia	22 (20.0)	75 (22.3	0.62	1.13 (0.64, 2.00)	0.66
UTI	19 (17.3)	33 (9.8)	0.034	0.50 (0.26, 0.96)	0.038

We adjusted for age, sex, race, ethnicity, insurance status, cancer, malnutrition, ED admission, and BMI in kg/m^2^

*LOS* Length of stay, *ICU* Intensive care unit, *IRR* Incident rate ratio, *OR* Odds ratio, *UTI* Urinary tract infection, *CI* Confidence interval; and *4-OLE* SMOFLipid

^*^Intralipid—(Baxter Inc, Deerfield, IL, USA)

## Discussion

This study is among the first to examine a switch to a soybean oil-sparing LE strategy in PN patients to demonstrate significant clinical benefits after the successful widespread implementation of 4-OLE in a hospital-wide fashion. Our data show the use of 4-OLE was associated with a shorter hospital LOS in both critically ill and all hospitalized patients and a shorter ICU LOS. The switch to 4-OLE from *Intralipid* (Baxter Inc, Deerfield, IL, USA) also demonstrated a significant reduction in total bilirubin from baseline levels in ICU patients and reduced TG levels in all adult patients. The switch to 4-OLE was also found to lead to a reduced risk of UTIs in the critically ill cohort.

These data are also among the first to describe a successful switch to a soybean oil-lipid sparing strategy using 4-OLE in a US hospital system. The use of a soybean oil-sparing strategy for PN regimens has long been advocated to limit the adverse effects associated with traditional lipid emulsions [6, 16, 17]. However, evidence describing outcomes and benefits of a full-scale switch in all hospitalized adult PN patients (not just ICU patients or higher-risk PN patients) use have not previously been described in a US healthcare setting. This study is the first to demonstrate significant benefits of a full-scale switch to a soybean oil-LE sparing strategy in a hospital setting. These data would support a soybean oil-LE sparing strategy in all hospitalized adult PN patients using 4-OLE.

Specifically, a switch to 4-OLE use was associated with reductions in hospital LOS and ICU LOS. Previous studies have reported a trend toward a decrease in LOS when soybean oil-sparing LE strategies have been employed, with some evidence suggesting that a reduced omega-6 LE content reduces ICU LOS [20]. Our data are consistent with this very recent meta-analysis of omega-6 sparing parenteral lipid emulsions showing substantial hospital LOS reductions [6 studies; weighted mean difference (WMD) − 6.88; 95% CI − 11.27, − 2.49; *p* = 0.002] in patients receiving an omega-6 sparing strategy [20]. Specific to fish oil (FO) containing lipid emulsions which increase EPA + DHA delivery, such as 4-OLE examined in our study, this same meta-analysis found FO-containing PN reduced the length of intensive care (8 studies; WMD − 3.53; 95% CI − 6.16, − 0.90; *p* = 0.009) as seen in our real-world hospital data. In these data, the use of 4-OLE was provided for both a significantly earlier hospital discharge and ICU discharge with no significant differences in readmission rates at 30 and 90 days. Although hospital costs could not be evaluated in this study, it can be expected that the reduced hospital and ICU LOS associated with a switch to 4-OLE use may also be associated with a reduction in costs related to reduced LOS [29].

We also observed some benefits of a soybean oil-LE sparing strategy with 4-OLE on infectious complications in our analysis. The literature has long reported the associated complication risks related to traditional LE. These are largely due to the pro-inflammatory and immune system hampering effects of its high omega-6 fatty acid composition [7–9]. Thus, traditional LE has been associated with infectious complications ranging from bacteremia to profound sepsis [11–13]. The results of 4-OLE use in this study demonstrated a reduced incidence of UTIs, specifically in the ICU population. Previous literature has reported a reduction in septic complications by up to 56% when soybean oil-sparing LE enriched with EPH + DHA has been used [27]. Our data are also consistent with the aforementioned very recent meta-analysis of omega-6 sparing parenteral lipid emulsions in ICU patients where the risk of infectious complications was reduced in ICU patients receiving soybean oil-sparing LE with added EPA + DHA (4 studies; RR 0.65; 95% CI 0.44, 0.95; *p* = 0.03).

4-OLE patients in this study also showed no difference in LE associated hepatic dysfunction. We observed no significant elevations in liver function parameters in both critically ill and all adult patients, except for minor elevations of ALP in this all adult cohort. Given that observed ALP elevation was isolated with no concomitant rise in AST, ALT, or total bilirubin levels, this elevation could be explained by bile stasis commonly seen in PN patients without associated hepatosteatosis [30]. In contrast, patients post-switch to 4-OLE were found to have improved TG and reductions in elevation of total bilirubin levels in a cohort with more extensive liver and renal disease.

PN-associated hepatic dysfunction in the adult population can vary from benign elevations to causes such as sepsis-related cholestasis [31]. However, the hepatic dysfunction with traditional soybean oil LE can manifest as hypertriglyceridemia which can increase pancreatitis risk as well as potential increased risk of mortality, particularly in prolonged use [31, 32]. Typical strategies to combat this dysfunction involve reducing lipid infusion rates which often create a more significant calorie debt in already malnourished patients or a need for increased glucose delivery, which can be associated with worsened hyperglycemia and adverse outcomes [32]. Thus, the observed effects of 4-OLE on mitigating hepatic dysfunction in this study may help avoid these potential complications and can again be explained by the mixed composition of the LE, which has been proven to lower the risk of liver dysfunction [33]. Recent lipid dosing guidelines have called for a recommendation of 1–2 g/kg/d delivery for 4-OLE formulations [34]. In the period when our data were collected, we had agreed to continue a similar lipid dosing strategy that had been utilized when we previously used the traditional soybean LE to assess safety and outcomes of this new lipid formulation in our hospital. Given the data presented here for safety and signals of improved clinical outcomes, we have begun to increase our clinical 4-OLE delivery closer to 1 g/kg/d in most patients. It is possible that increasing this dose and thereby delivering additional fish oil (and importantly EPA/DHA) may further improve clinical outcomes and potentially show additional benefit in reducing elevations in liver function measures. Further studies will be needed to explore this.

Overall, the observed results in this study provide further evidence supporting a switch to soybean oil-LE sparing strategy using 4-OLE in all hospitalized adult PN patients, and not just selected groups (i.e., ICU or high risk patients). Our data demonstrating the successful implementation of 4-OLE hospital-wide are the first of its kind, and this analysis demonstrates that this switch to a soybean oil-LE sparing strategy is associated with safety and clinical benefit. The hospital-wide switch to 4-OLE reduced overall and ICU LOS compared to traditional IL use. Its use also significantly reduced the risk of UTIs and hepatic dysfunction, with decreased TG and total bilirubin levels in a hospital-wide patient population. Overall, the findings in this study prove a switch to a soybean oil-LE sparing strategy using a 4-OLE with additional EPA + DHA is feasible and safe and is associated with improved clinical outcomes in adult PN patients.

### Limitations

This study has a number of limitations inherent to a retrospective before-/after-observational study. First, we do not have data regarding the specific characteristics leading to PN indications and contraindications to enteral feeding. Second, we do not know from our data if patients were fed simultaneously via oral or EN while receiving PN. In relation, we do not have information regarding interruptions or duration of PN in both cohorts, which could alter the interpretation of our findings. Lastly, given that this study was conducted in one isolated hospital system, the generalizability to a national population is limited. However, the results demonstrate significant value as it is the first to demonstrate clinical benefit with widespread 4-OLE implementation.

## Conclusion

The use of soybean oil-sparing strategies for LE support in those receiving PN has long been advocated for with limited available evidence. 4-OLE use, though relatively new, was successfully implemented in a large institution. Switch to 4-OLE was associated with reduced LOS, complications, and mitigated hepatic dysfunction in critically ill adult patients. The findings in this study suggest that 4-OLE use is efficacious and safe and may improve clinical outcomes among patients requiring PN.

## Data Availability

All raw data are available upon request.
